# Effects of ventilator settings, nebulizer and exhalation port position on albuterol delivery during non-invasive ventilation: an in-vitro study

**DOI:** 10.1186/s12890-016-0347-5

**Published:** 2017-01-10

**Authors:** Yuda Sutherasan, Lorenzo Ball, Pasquale Raimondo, Valentina Caratto, Elisa Sanguineti, Federico Costantino, Maurizio Ferretti, Robert M. Kacmarek, Paolo Pelosi

**Affiliations:** 1 IRCCS AOU San Martino-IST, Department of Surgical Sciences and Integrated Diagnostics, University of Genoa, Genoa, Italy; 2 Division of pulmonary and critical care medicine, Faculty of medicine, Ramathibodi Hospital, Mahidol University, Bangkok, Thailand; 3 Dipartimento di Anestesia, Rianimazione e Terapia Intensiva, Università degli Studi di Foggia, Foggia, Italy; 4 Department of Chemistry and Industrial Chemistry, University of Genoa, Genoa, Italy; 5 SPIN-CNR, Genoa, Italy; 6 Department of Anesthesiology, Harvard Medical school, Department of Anesthesiology, Critical Care and Pain Medicine, and the Department of Respiratory Care, Massachusetts General Hospital, Boston, Massachusetts USA

**Keywords:** Aerosol, Bronchodilators, Albuterol, Continuous positive airway pressure, CPAP, NIV, BIPAP, Nebulizers

## Abstract

**Background:**

Few studies have investigated the factors affecting aerosol delivery during non-invasive ventilation (NIV). Our aim was to investigate, using a bench-top model, the effect of different ventilator settings and positions of the exhalation port and nebulizer on the amount of albuterol delivered to a lung simulator.

**Methods:**

A lung model simulating spontaneous breathing was connected to a single-limb NIV ventilator, set in bi-level positive airway pressure (BIPAP) with inspiratory/expiratory pressures of 10/5, 15/10, 15/5, and 20/10 cmH_2_O, or continuous positive airway pressure (CPAP) of 5 and 10 cmH_2_O. Three delivery circuits were tested: a vented mask with the nebulizer directly connected to the mask, and an unvented mask with a leak port placed before and after the nebulizer. Albuterol was collected on a filter placed after the mask and then the delivered amount was measured with infrared spectrophotometry.

**Results:**

Albuterol delivery during NIV varied between 6.7 ± 0.4% to 37.0 ± 4.3% of the nominal dose. The amount delivered in CPAP and BIPAP modes was similar (22.1 ± 10.1 vs. 24.0 ± 10.0%, *p* = 0.070). CPAP level did not affect delivery (*p* = 0.056); in BIPAP with 15/5 cmH_2_O pressure the delivery was higher compared to 10/5 cmH_2_O (*p* = 0.033) and 20/10 cmH_2_O (*p* = 0.014). Leak port position had a major effect on delivery in both CPAP and BIPAP, the best performances were obtained with the unvented mask, and the nebulizer placed between the leak port and the mask (*p* < 0.001).

**Conclusions:**

In this model, albuterol delivery was marginally affected by ventilatory settings in NIV, while position of the leak port had a major effect. Nebulizers should be placed between an unvented mask and the leak port in order to maximize aerosol delivery.

## Background

Non-invasive positive pressure ventilation (NIV) is being used increasingly in patients with either acute or chronic respiratory failure [1]. The use of NIV as first line treatment for acute exacerbations of chronic obstructive pulmonary disease (COPD) have been confirmed by several studies [2, 3] that showed a reduction of complications, frequency of intubation and mortality in patients with hypercapnic respiratory failure. Although cost-effectiveness of long term NIV remains debated [4], in specific subgroups of COPD patients such as obese [5] or severely ill patients [6], long term domiciliary home ventilator NIV improves survival. In these patients, nebulized bronchodilators are often used to relieve airway obstruction; however, few studies have reported the factors affecting aerosol delivery during NIV and most of the studies focused on intubated patients [7–9]. Previous studies showed that the albuterol delivery, namely the amount that effectively reaches the patients’ airways, is affected by the bi-level positive airway pressure (BIPAP) ventilatory settings: respiratory rate, inspiratory positive airway pressure (IPAP) and end expiratory positive airway pressure (EPAP) [10]. NIV can be delivered through several interfaces: the most commonly used oro-nasal and nasal masks have a built-in leak port, but this design resulted in reduced albuterol delivery in in-vitro studies [11, 12]. Conflicting results have been reported concerning the role of the nebulizer and leak port position with unvented masks [13, 14]. In a recent study, CPAP administered with an high-flow system resulted in low drug delivery rates; higher flows and lower CPAP levels were associated with worse performances [15], but these findings cannot be translated directly to patients receiving CPAP through a NIV ventilator.

Our goal was to investigate, with a bench-top model, the effect of different positions of the exhalation port and nebulizer during CPAP or BIPAP administered with a dedicated NIV ventilator on aerosol delivery. We tested masks with and without built-in exhalation ports, using common ventilator settings. We hypothesized that albuterol delivery by nebulization during NIV is affected by the positions of the exhalation port and pressure levels.

## Methods

### Experimental settings

**Fig. 1:**
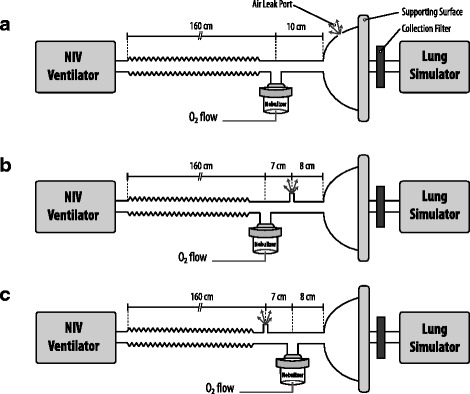
Experimental settings, see text for detailed explanation. Configuration **a**: leak port included in the vented mask; Configuration **b**: leak port between the nebulizer and the unvented mask; Configuration **c**: leak port between the nebulizer and the respiratory circuit

A pneumatic lung simulator system (Dimar, Mirandola, Italy) was set to mimic a patient with mild tachypnea with a tidal volume of 400 mL, respiratory rate of 20 cycles per minute, and inspiratory to expiratory ratio of 1:2. As illustrated in Fig. 1, the lung simulator was connected, through a corrugated tube, to a rigid holed surface where the tested masks were firmly fixed in order to avoid air leaks from the mask seal. The masks were connected to a Puritan Bennett 560 non-invasive ventilator for domiciliary use (Covidien, Dublin, Ireland), with a single-limb ventilatory circuit. The ventilator was set in CPAP or BIPAP mode, in the latter case with a rise time of 0.2 s and the inspiratory trigger sensitivity set at −2 L/m.

The following commonly used ventilator settings were tested: BIPAP mode, IPAP/EPAP of 10/5, 15/10, 15/5, and 20/10 cmH_2_O; CPAP with pressure levels of 5 and 10 cmH_2_O. Two oro-nasal masks were tested: a vented mask incorporating a leak port (Respireo Primo F vented, Air Liquide, France), and an unvented mask (Respireo Primo F unvented, Air Liquide, France), with a single-arch leak port (Respironics, Pennsylvania, US) in the ventilator circuit. For a more detailed description of the respiratory circuits, see Fig. 1. Nebulization was obtained with a small volume nebulizer (Teleflex Hudson RCI, California, US), filled with 3 ml of distilled water containing 5 mg of albuterol, a clinically effective dose to relieve bronchospasm [16] and used in previous bench studies [11], and driven with 8 L/m of oxygen, according to the manufacturer’s recommendations. The nebulizer was operated in the vertical position, for 10 min, time in which the nebulizer chamber was empty. Each experimental condition was tested in three replicates.

### Collection filter and albuterol detection

A custom-made collection filter, described in a previously published study, was interposed between the mask and the lung simulator, to collect the albuterol delivered to the lung simulator [15]. A commercially available ventilator circuit filter (DAR Sterivent, Covidien, Dublin, Ireland) was opened, its hydrophobic membrane removed and replaced with 34 cm^2^ absorbent paper. At the end of each trial, the filter was opened. Ten milliliters of distilled water were used to wash the paper and the internal plastic surface of the scaffold to extract albuterol. The solution was then stirred for one minute with a vortex oscillator (ZX3, VELP Scientifica, Monza, Italy). Each lavage solution was transferred to a 1 ml quartz cuvette, and its absorbance at 276 nm was measured with a spectrophotometer (Lambda 35, Perkin Elmer, Waltham, US). This method was adapted from the current literature [13, 17, 18] and previously described.

### Statistical analysis

The amount of delivered albuterol was defined as percent of the dose initially placed in the nebulizer that was trapped by the collection filter, calculated with the following formula: (μg of albuterol detected/5,000 μg) × 100. From a previous study [15], we expected an inter-experimental standard deviation of 0.5%. Therefore, repeating each configuration test in three replicates would have provided 80% power (1- β) to detect, with a two-tailed α of 0.05, differences with an effect size of at least 2.8, corresponding to a 1.4% difference in albuterol delivery. The effect of CPAP, IPAP, EPAP and position of the leak port on the percent of delivered albuterol were assessed with a general linear model, with Sidak post-hoc correction for multiple comparisons. The statistical analysis was performed using SPSS version 21 (IBM, Corp., Chicago, Illinois, US). Data were reported as mean ± standard deviation, and statistical significance was considered for *p* < 0.05.

## Results

Albuterol delivery ranged between 6.7 ± 0.4 to 37.0 ± 4.3% of the nominal dose. Table 1 resumes the albuterol delivery in all configurations. The average albuterol delivery in all BIPAP configurations did not differ from the average delivery during CPAP (22.1 ± 10.1 vs. 24.0 ± 10.0%, respectively, *p* = 0.070).

**Table 1:** Albuterol delivery in each configuration

	Airway Pressure (cmH_2_O)		Albuterol delivery (% of dose)			
Mode	EPAP or CPAP	IPAP	Config. A	Config. B	Config. C	Pairwise comparison (between configurations)
CPAP	5	-	12.5 ± 0.4	26.4 ± 3.6	37.0 ± 4.3	*p* < 0.001
	10	-	11.5 ± 1.7	23.9 ± 1.5	32.8 ± 2.6	*p* < 0.001
BIPAP	5	10	9.5 ± 1.0	22.1 ± 3.5	30.9 ± 1.5	*p* < 0.001
	5	15	13.5 ± 3.6*	27.7 ± 1.9*	35.1 ± 4.0*	*p* < 0.001
	10	15	6.7 ± 0.4	25.2 ± 1.1	34.1 ± 3.6	*p* < 0.001
	10	20	8.7 ± 0.9	25.7 ± 2.2	26.5 ± 6.1	*p* < 0.001

*CPAP*, Continuous Positive Airway Pressure, *BIPAP* Bilevel Positive Airway Pressure, *EPAP* Expiratory Positive Airway Pressure, *IPAP* Inspiratory Positive Airway Pressure

*Significantly higher compared to BIPAP 10/5 cmH_2_O and BIPAP 20/10 cmH_2_O (*p* < 0.05)

**Fig. 2:**
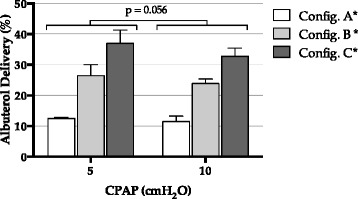
Albuterol delivery during CPAP. See text for detailed description of configurations *A*, *B* and *C*. *Pairwise comparisons between configurations all *p* < 0.001

**Fig. 3:**
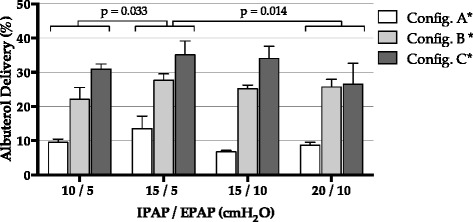
Albuterol delivery during BIPAP. See text for detailed description of configurations *A*, *B* and *C*. *Pairwise comparisons between configurations all *p* < 0.001

In CPAP, pressure level marginally affected the amount of delivered albuterol (*p* = 0.056), as illustrated in Fig. 2. In BIPAP, the configuration with IPAP/EPAP of 15/5 cmH_2_O delivered higher albuterol amounts compared to 10/5 cmH_2_O (*p* = 0.033) and 20/10 cmH_2_O (*p* = 0.014), as shown in Fig. 3.

In both CPAP and BIPAP, the most relevant differences were observed changing the leak port position (*p* < 0.001 in all pairwise comparisons), with the vented mask (Fig. 1a) resulting in the lowest delivery, and the unvented mask with the nebulizer placed between the mask and the leak port (Fig. 1c) resulting in the highest delivery (Figs. 2 and 3).

## Discussion

The main findings of this in-vitro study are: 1) the amount of the delivered albuterol varied between 6.7 and 37% of the nominal dose and it was comparable in CPAP and BIPAP mode; 3) the highest albuterol delivery was obtained with the nebulizer placed between the leak port and the non-vented mask; 4) there was no difference in albuterol delivery between the two tested CPAP levels; 5) In BIPAP, ventilator settings marginally affected albuterol delivery, with the best performances obtained with 15/5 cmH_2_O IPAP/EPAP.

The use of NIV has gained popularity in the last decades, becoming the first line therapy for patients presenting with COPD, both during acute respiratory failure and post discharge using dedicated ventilators intended for domiciliary use [19]. Inhaled bronchodilators are always part of the treatment during exacerbation and long-term management of these patients. Although there are a number of studies addressing inhaled drug administration during invasive mechanical ventilation [20–22], few data are available focusing on aerosol therapy during application of NIV [12, 14]. Since home BIPAP ventilators typically have a single limb respiratory circuit, there are intrinsic differences with the two-limb circuits used on intensive care ventilators. Therefore, the findings of studies using critical care ventilators cannot be translated directly to this setting. The presence of an intentional leak port in the circuit creates a bias flow towards the exterior, potentially affecting drug delivery.

Our findings confirm what was previously described by Chatmongkolchart et al., that reported an increased aerosol delivery with the nebulizer placed between the mask and the leak port during BIPAP [14]. A recent study by Dai et al. [13] investigated the influence of different types of exhalation valves and nebulizer positions, finding an opposite result for most of the tested configurations, reporting better delivery when the nebulizer was placed between the NIV ventilator and the leak port. Dai et al. studied different types of exhalation valves, including the type used in our study. With such single-arch valve, the role of nebulizer position was puzzling and the best position varied at different ventilation settings. In our experimental setting the advantage of configuration C was observed at all ventilator settings, confirming the findings of a previous study [14] and extending it to CPAP ventilation.

During NIV, the exhalation port is incorporated within the circuit or in the mask in order to allow gas washout during exhalation. With a non-vented mask and the nebulizer placed between the mask and the leak port, the inspiratory pressure moves aerosol droplets to the patient. During exhalation aerosol leaks from the exhalation port, with a retrograde shift of aerosol droplets, potentially accumulating in the tubing with the tubing acting as a reservoir for delivery during the next inspiration. On the other hand, when the nebulizer is located between the leak port and the NIV ventilator, aerosol leakage occurs both in the inspiratory and early expiratory phases [14]. With a non-vented mask and the nebulizer placed before the leak port, or with the leak port incorporated in the mask, leakage occurs during both inspiration and expiration causing a significant decrease of aerosol delivery.

In BIPAP mode, higher EPAP resulted in a slight decrease in bronchodilator delivery. Higher EPAP, causes a higher retrograde flow during expiration with leakage of aerosol through the leak port, as described previously [14]. The IPAP/EPAP resulting in the highest aerosol delivery was 15/5 cmH_2_O. However, all ventilator pressure settings only resulted in minor changes in the delivery efficiency, especially when compared to the major effect due to the position of the leak port.

This is the first study investigating albuterol delivery during CPAP delivered with a single limb NIV ventilator. In a recent study [15], aerosol delivery was studied during high-flow Venturi-based CPAP administration: efficiency of aerosol delivery was affected by CPAP levels and driving flow, and was low, with an average of 14% for the best performing respiratory circuit. The absolute value of drug delivery in our study was comparable to those reported in the few previous studies with BIPAP [13, 14], but higher than the study investigating high-flow CPAP [15]: in our present study, CPAP delivered through a NIV ventilator resulted in higher delivery rates, above 30% at both 5 cmH_2_O and 10 cmH_2_O CPAP. This could be due to the fact that, in high flow systems, flow itself is used to generate pressure resulting in a high bias flow through the CPAP valve, with a washout effect on aerosol droplets. Differently, NIV ventilators automatically adjust output flow to achieve the desired pressures, therefore net flow from the respiratory circuit to the ambient is lower. On the other hand, this lack of a significant circuit leak flow, along with the single-limb circuit design, is what makes the presence of a leak port necessary. The position of the leak port in relationship to the nebulizer was found to be the most determinant factor influencing albuterol delivery, both in CPAP and BIPAP modes.

Our experimental study has some limitations. We did not investigate the effect of humidification, a known factor affecting delivery [23]. We used two identical masks, differing only for the presence of an incorporated leak port. Therefore, our results cannot be directly generalized to other devices, including different types of interface, leak ports and nebulizers using different mechanisms of action [24]. Albuterol was nebulized continuously during both inspiration and expiration: we would expect different results with nebulizers delivering aerosol only in the inspiratory phase. Moreover, only few commonly used pressure levels were tested, in particular our study does not provide information concerning higher inspiratory pressures, that however are seldom used in the clinical practice. The findings concerning leak port position and ventilator settings should not be influenced by changes in these factors. Since the clinical efficacy of aerosol inhalation depends on the aerosol deposition in the airways, which is affected by the ventilation mode, as well as the dead space and aerosol particles penetration in the respiratory system, further studies in-vivo are warranted to clarify these effects in terms of clinical efficacy and side effects particularly in patients with type two respiratory failure.

## Conclusions

In the present in-vitro model, the single factor most affecting aerosol delivery during NIV or non-invasive CPAP delivered by a single circuit was the position of the nebulizer and leak port. Drug delivery is greatest with the nebulizer placed between the leak port and a non-vented mask, regardless of ventilator settings.
